# Oral Microbiota of Children Is Conserved across Han, Tibetan and Hui Groups and Is Correlated with Diet and Gut Microbiota

**DOI:** 10.3390/microorganisms9051030

**Published:** 2021-05-11

**Authors:** Ke Liu, Siyu Chen, Jing Huang, Feihong Ren, Tingyu Yang, Danfeng Long, Huan Li, Xiaodan Huang

**Affiliations:** 1 School of Public Health, Lanzhou University, No. 222 Tianshuinanlu, Lanzhou 730000, China; angelilk@163.com (K.L.); hayx19@lzu.edu.cn (S.C.); jhuang17@lzu.edu.cn (J.H.); renfh20@lzu.edu.cn (F.R.); yangty20@lzu.edu.cn (T.Y.); Longdf@lzu.edu.cn (D.L.); 2 Henan Provincial Center for Disease Control and Prevention, Nongye East Road, Zhengzhou 450000, China

**Keywords:** host-microbe interactions, salivary microbiota diversity, gut microbiota, dietary habit, ethnicity, Qinghai–Tibetan plateau

## Abstract

The oral microbiota can be affected by several factors; however, little is known about the relationship between diet, ethnicity and commensal oral microbiota among school children living in close geographic proximity. In addition, the relationship between the oral and gut microbiota remains unclear. We collected saliva from 60 school children from the Tibetan, Han and Hui ethnicities for a 16S rRNA gene sequencing analysis and comparison with previously collected fecal samples. The study revealed that *Bacteroidetes* and *Proteobacteria* were the dominant phyla in the oral microbiota. The Shannon diversity was lowest in the Tibetan group. A PCA showed a substantial overlap in the distribution of the taxa, indicating a high degree of conservation among the oral microbiota across ethnic groups while the enrichment of a few specific taxa was observed across different ethnic groups. The consumption of seafood, poultry, sweets and vegetables was significantly correlated with multiple oral microbiotas. Furthermore, 123 oral genera were significantly associated with 191 gut genera. A principal coordinate analysis revealed that the oral microbiota clustered separately from the gut microbiota. This work extends the findings of previous studies comparing microbiota from human populations and provides a basis for the exploration of the interactions governing the tri-partite relationship between diet, oral microbiota and gut microbiota.

## 1. Introduction

Oral health is considered to be a fundamental component of general health and poor oral health such as dental caries and periodontitis can lead to pain, poor nutrition and high treatment costs ultimately decreasing the quality of life [1]. In particular, children are in a critical developmental period when their permanent teeth replace their deciduous teeth and some oral diseases can affect the development of permanent teeth [2]. Therefore, the identification of risk factors in children can help reduce the prevalence of oral diseases that have been shown to have a clear association with the oral microbiota [3]. The oral cavity contains the second most complex microbiota in the human body after the colon [4,5] and includes approximately 700 species, most of which are indigenous [6]. Abundant evidence supports the direct and indirect influence of the oral microbiota on the formation and development of oral diseases such as dental caries and periodontal disease [7,8]. Additionally, oral microbial communities reportedly participate in several systemic diseases such as cancer, pneumonia, preterm low birth weight, atherosclerosis and coronary heart disease [9,10,11,12,13].

The composition of the oral microbiota is highly variable from person to person and is affected by common factors including diet, the timing of food consumption, drinking and smoking [14,15,16,17]. Among individuals living in different geographical areas with marked environmental differences, variations in the composition of the oral microbiota have been observed among ethnicities [18,19]. However, it remains unclear whether and how ethnicity contributes to the structure of the oral microbiota among people living in close proximity with shared environmental factors. Furthermore, a few gaps remain in the current understanding of how diet can impact on the oral microbiota. We have previously reported on the gut microbial diversity of school children from the Qinghai–Tibetan Plateau and found that the dietary structure was associated with specific gut microbial taxa [20]. In addition, previous studies have reported a relationship between the oral microbiota and the structure of the gut microbiota [21]. Prior studies found that 1.5 × 10^11^ bacterial cells flow from the mouth to the stomach per day [22], suggesting that the oral microbiota may potentially directly affect the gut microbiota. Given the possibility of this influence, and established knowledge that the gut microbiota participates in nutrient metabolism, immunomodulation and gut barrier maintenance [23,24,25,26], a detailed correlation analysis between the oral microbiota, diet and the gut microbiota is thus warranted. In light of our previous data on diet-related differences in the gut microbiota from Han, Hui and Tibetan children living in close proximity, we proposed that the composition and diversity of the oral microbiota was related to diet, which were both related to the composition and diversity of the gut microbiota.

In the present study, we investigated the oral microbiota in salivary samples obtained from 60 Tibetan, Han and Hui school children living in the Qinghai–Tibetan Plateau using a 16S rRNA analysis. The purpose of this study was to address three primary questions regarding the oral microbiota in healthy school children: (1) Is the diversity of the oral microbiota different among the three ethnic groups? (2) What is the relationship between diet and the oral microbiota? (3) Is there a correlation in the diversity and composition between the oral and gut microbiota?

## 2. Materials and Methods

### 2.1. Ethic Approval and Consent to Participate

All procedures performed were approved by the Medical Ethics Committee of the School of Public Health in Lanzhou University (No. 20170227-2). Participants received detailed information about the study and informed consent was obtained from guardians and the administrator/teacher of Labrang Primary School.

### 2.2. Subject Selection and Sample Collection

A total of 60 school children (9.30 ± 0.87 years) from the Labrang Primary School, which is located on the Qinghai–Tibetan Plateau in Xiahe County, Gannan Tibetan Autonomous Prefecture (see the map in Supplementary File 1, 3200 m above sea level), were recruited for the study. The average body mass index of the children was 15.56 ± 2.20 kg/m^2^. All subjects were divided into three groups according to ethnicity: the Han group (n = 20, 8 males and 12 females), the Tibetan group (n = 20, 11 males and 9 females) and the Hui group (n = 20, 12 males and 8 females). Saliva from all participants was sampled at home on the morning of 21 September 2017. Unstimulated saliva was expectorated into a sterile tube before eating breakfast and brushing teeth. Finally, the samples were maintained in liquid nitrogen immediately, transported to the laboratory in liquid nitrogen (5 h) and stored at −70 °C before a further analysis. The gut microbial data information of 60 subjects were obtained from the European Nucleotide Archive (http://www.ebi.ac.uk/ena/data/view/PRJEB30788, accessed on 15 January 2019) as per our previous research, which was carried out together with the current study [20]. A dietary survey was conducted with the assistance of the undergraduates from Lanzhou University. Food categories from questionnaire included grains, vegetables, fruits, poultry meat, livestock meat, seafood, dairy, beans, eggs, nuts, condiments and sweets. The dietary frequency questionnaires were adopted to obtain the intake of each food that every child had consumed in the past six months. CDGSS3.0 nutrition software (according to the China Food Composition Database by the Chinese Center for Disease Prevention and Control) was used to analyze the nutritional intake of each subject. All children did not take antibiotics within the last three months before sampling.

### 2.3. DNA Extraction

Total DNA extraction from the saliva samples was carried out using a QIAamp DNA stool mini kit (QIAGEN, Hilden, Germany) according to the manufacturer’s instructions. Agarose gel electrophoresis was used to evaluate the integrity of the purified DNA and the electrophoresis bands were clearly visible without degradation. The DNA concentration and quality were determined by using a Nanodrop 2000 (Thermo Fisher Scientific, Waltham, MA, USA). All DNA samples were stored at –20 °C until required for a further analysis.

### 2.4. Polymerase Chain Reaction Amplification and Illumina MiSeq Sequencing

For the analysis of the oral microbiota, the V3-V4 hypervariable regions of the bacterial 16S rRNA gene were amplified using primers 341F (5′-CCTACGGGNGGCWGCAG -3′) and 805R (5′-GACTACHVGGGTATCTAATCC-3′) in a polymerase chain reaction (PCR) on an ABI 2720 Thermal Cycler (Thermo Fisher Scientific, USA). The method for the gut microbiota analysis was described in a previous study [20]. The reactions were performed in triplicate for each sample. The PCR program was as follows: initial denaturation (95 °C, 3 min), 27 cycles of denaturation (95 °C, 30 s), annealing (55 °C, 30 s), elongation (72 °C, 45 s) and a final extension (72 °C, 10 min). The final PCR products were quantified with an Agilent 2100 Bioanalyzer (Agilent Technologies, Santa Clara, CA, USA) and purified with AMPure XP beads (Beckman Coulter, Brea, CA, USA) according to the manufacturer’s protocol. The sequencing was conducted using a MiSeq Reagent Kit v3 (Illumina, San Diego, CA, USA). The raw reads were deposited by accession number PRJEB34215 into the European Nucleotide Archive (http://www.ebi.ac.uk/ena/data/view/PRJEB34215, accessed on 1 September 2019).

### 2.5. 16S rRNA Gene Analysis

TrimGalore, FLASH2, mothur and Usearch software were used for trimming, primer removal and filtering based on length. After filtering, the qualified reads were clustered into operational taxonomic units (OTUs) with a 97% similarity. The Ribosomal Database Project Classifier [27] was used to annotate the taxonomic information at an 80% confidence threshold. The alpha diversities (Shannon diversity and observed species) of the bacterial communities among groups were calculated by mothur software. To assess the beta diversity, a principal coordinate analysis (PCoA) based on the unweighted UniFrac and weighted UniFrac was performed for the visualization of the microbiome structure separation across groups by R software (version v3.5.1). A Venn diagram [28] constructed again using R was used for the identification of the core microbiome at the OTU level. The rarefaction curves [29] were evaluated based on the observed species.

### 2.6. Statistical Analysis

The alpha diversities of the three groups were compared using the Kruskal–Wallis rank sum test. The differences in the relative abundance of the taxonomic units between the groups were tested by one-way analysis of variance (ANOVA). Spearman’s rank correlation was used to test whether the alpha diversities of the oral microbial communities were significantly associated with those of the gut microbial communities. A permutational multivariate analysis of variance (PERMANOVA) based on unweighted UniFrac distances was performed to reveal the differences and similarities in the microbiota among groups using R software (version v3.4.3). The correlation between the oral microbiome and environment factors or the gut microbial communities was evaluated using Pearson’s correlation. CDGSS 3.0 nutrition software was used for the analysis of the food intake of each subject every day. Statistical analyses were conducted by using SPSS (version 22.0). *p* values < 0.05 indicated a statistical significance.

## 3. Results

### 3.1. Sequence Information

Illumina MiSeq sequencing of saliva samples yielded 5,322,029 reads. A total of 4,600,398 (86.4%) clean reads passed quality control and filtering for subsequent analyses. An average of 76,673 sequences were obtained from each sample. The mean sequence length was 421.50 bp, ranging from 100 bp to 452 bp. We thus obtained a total of 30,672 OTUs from the qualified sequences at a 97% similarity. The rarefaction curves reached a plateau, suggesting that the depth of the sequencing was adequate to capture the full taxonomic diversity within each sample (Supplementary Figure S1).

### 3.2. Oral Microbiota Are Largely Conserved among Ethnic Groups

**Figure 1:**
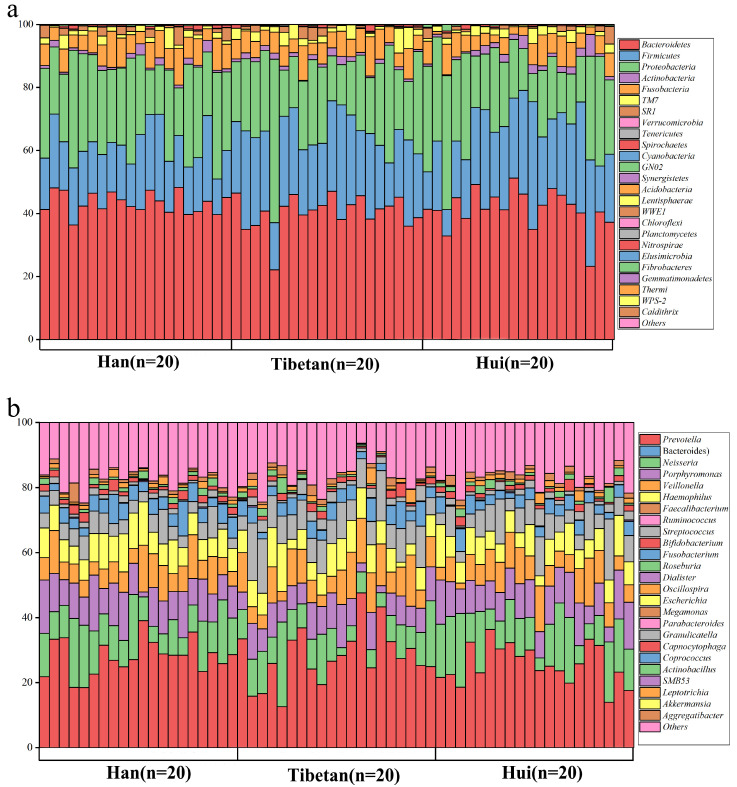

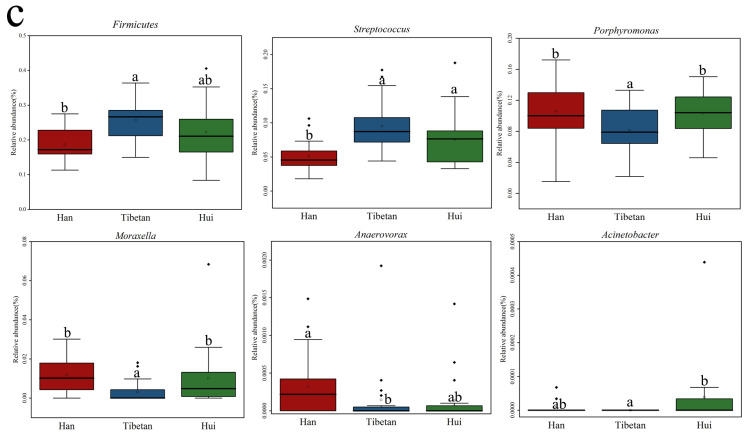
The composition of the oral microbiota at the (**a**) phylum and (**b**) genus levels. (**c**) The relative abundances of the significantly different taxa in the Han, Tibetan and Hui groups at the phylum and genus level. Different lower-case letters(a, ab, b,) indicate significant differences between the groups.

In order to investigate whether ethnicity could potentially affect the oral microbiota composition and structure, we sequenced the 16s rRNA in saliva to identify the bacterial taxa present in those samples, compared the composition of the oral microbiota and conducted diversity analyses. We detected members of 27 bacteria phyla although most sequences (96.58%) belonged to five phyla, namely, *Bacteroidetes* (mean relative abundance = 41.19%), *Proteobacteria* (24.83%), *Firmicutes* (22.14%), *Fusobacteria* (6.38%) and *TM7* (2.04%) (Figure 1a). At the genus level, *Prevotella* (27.22%), *Neisseria* (11.50%), *Porphyromonas* (9.70%), *Veillonella* (8.26%) and *Haemophilus* (7.66%) were the five dominant bacterial genera in the oral microbiota, occupying 64.34% of the whole (Figure 1b). An analysis by ANOVA revealed that the mean relative abundances of the majority of salivary microbiota were not significantly different among the three groups except for one phylum and five genera (Figure 1c). The mean relative abundance of *Firmicutes* was higher in the Tibetan population than in the Han population (*p* < 0.01). *Streptococcus* was significantly higher in the Hui population than in the Han population (*p* < 0.05). *Moraxella*, *Porphyromonas* and *Acinetobacter* were richer in the Hui group than in the Tibetan group while the Han group had a lower abundance of *Streptococcus* but higher *Moraxella*, *Anaerovorax* and *Porphyromonas* than the Tibetan group (*p* < 0.05).

**Figure 2:**
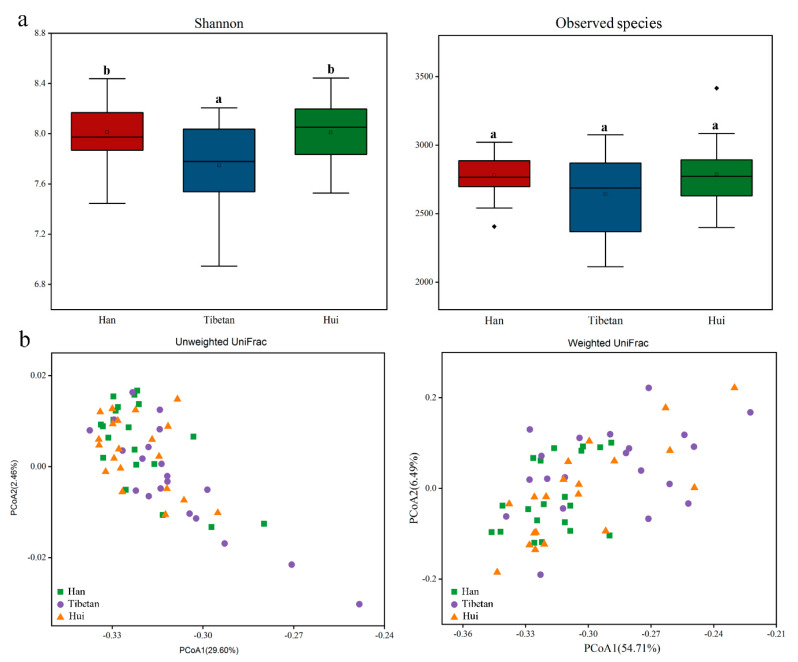
(**a**) Alpha diversity indexes (Shannon diversity and observed species) of the three ethnic groups. Different lower-case letters indicate significant differences between the groups. (**b**) PCoA based on unweighted and weighted UniFrac distances comparing the bacterial community among the three ethnic groups.

Alpha diversity indices including the Shannon diversity and observed species were calculated to identify the differences in the evenness and richness of taxa among all samples. A Kruskal–Wallis rank sum test was then used to compare the alpha diversity indices between the groups. The observed species showed no statistical differences across the three groups (*p* > 0.05). However, comparisons of the Shannon indexes revealed that the Tibetan group had the lowest diversity (*p* < 0.01) while the Hui and Han groups were not significantly different (Figure 2a). A PCoA showed a high degree of overlap in the distribution of samples from the three groups with none of the groups separating into individual clusters (Figure 2b). A PERMANOVA analysis also revealed that the three ethnic groups did not have a significant difference in diversity (*p* > 0.05).

We identified 18,818, 18,524 and 18,628 OTUs in the Han, Hui and Tibetan groups, respectively. The three ethnic groups shared 8838 OTUs, constituting 28.8% of all of the detected OTUs (Supplementary Figure S2). The shared OTUs were comprised of a stable and consistent core salivary microbiome. The OTUs that were not shared consistently among groups comprised the variable microbiome. Among these, 4856 OTUs were found only in the Han group, 4687 OTUs only in the Tibetan group and 4669 OTUs only in the Hui group.

### 3.3. Correlation between Oral Microbiota (Genus Taxon) and Dietary Intake

To better understand the correlation between the oral microbiota and diet, we assessed the dietary intake of the school children and subsequently conducted a correlation analysis with the microbiome data. The average daily intake of 12 specific foods for all participants is presented in Supplementary Table S1. In general, no significant differences were observed between the diets of the three ethnic groups with the exception of poultry consumption, which was significantly lower among Tibetans. No significant differences were observed between the total energy and energy proportions derived from protein, carbohydrate and fat macronutrients among the three ethnic groups (Supplementary Table S2).

**Figure 3:**
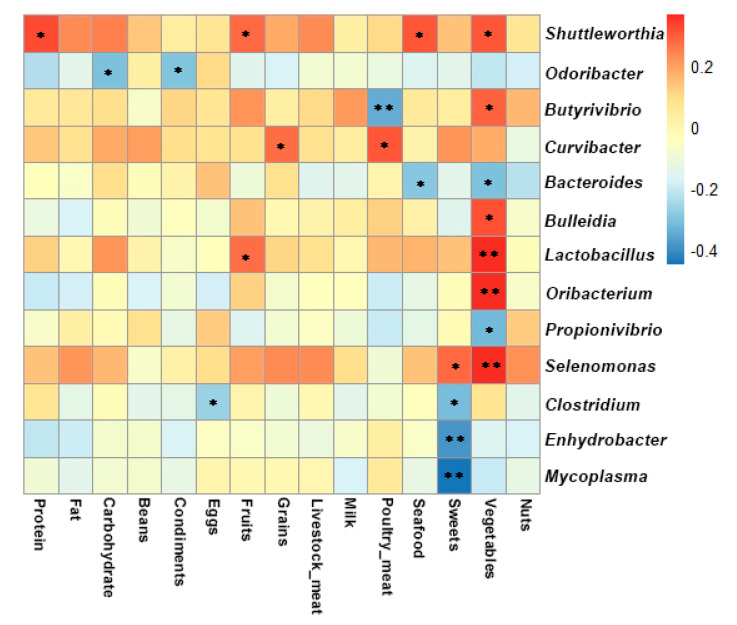
Genus level correlation heatmap between the oral microbiota and dietary intake (* *p* < 0.05, ** *p* < 0.01; the *p* value was calculated using the Pearson correlation).

A correlation analysis revealed significant relationships between specific foods and specific salivary bacteria at the genus level with a correlation coefficient of >0.3 or <–0.3 (Figure 3). The percentage of energy from protein and the consumption of seafood were all positively related with the relative abundance of *Shuttleworthia* (r = 0.31–0.32, *p* < 0.05). Poultry meat intake showed a negative association with *Butyrivibrio* (r = −0.35, *p* < 0.01) and a positive association with *Curvibacter* (r = 0.30, *p* < 0.05). *Clostridium*, *Enhydrobacter* and *Mycoplasma* were negatively associated with the intake of sweets (r = −(0.32–0.45), *p* < 0.05). *Bulleidia*, *Lactobacillus*, *Oribacterium*, *Selenomonas* and *Shuttleworthia* were positively correlated to vegetable intake (r = 0.30–0.36, *p* < 0.05) and *Bacteroides* and *Propionivibrio* were inversely related to vegetable intake (r = −0.32, *p* < 0.05).

### 3.4. Oral and Gut Microbial Communities Exhibit Both Differences and Correlations in Composition and Structure

In order to identify relationships and differences between the oral and gut microbiota, we compared their composition and structure and then used a Pearson correlation analysis to detect the correlations. The oral and gut microbiota of each participant had unique compositions (Supplementary Figure S3). Furthermore, the distributions of bacterial taxa differed between the gut and oral communities at both the genus and phylum levels. *Firmicutes* and *Bacteroidetes* were the most predominant phyla in the gut microbiomes whereas *Firmicutes* was less dominant in the gut. At the genus level, *Prevotella* and *Neisseria* were the predominant genera in the oral microbiota whereas *Bacteroides* and *Faecalibacterium* were the predominant genera in the gut.

**Figure 4:**
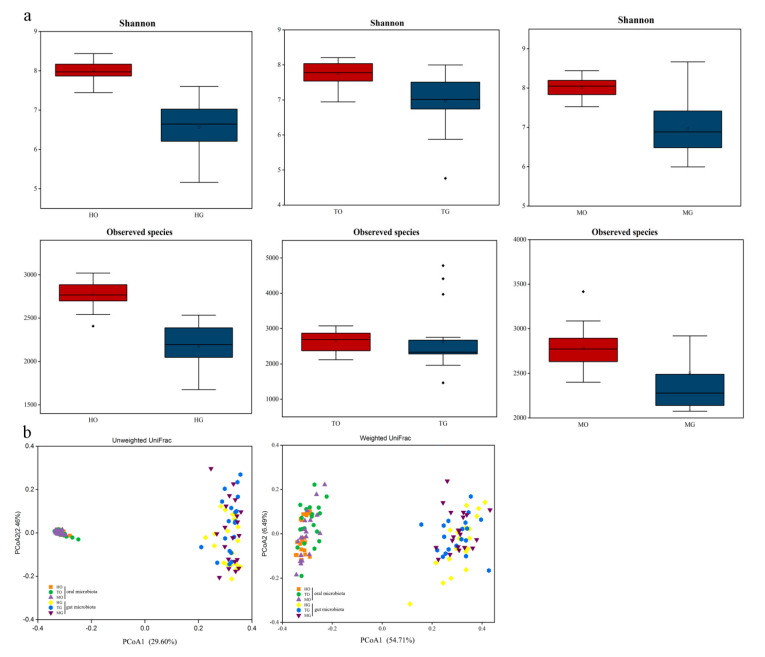
(**a**) Alpha diversity indices of the oral and gut microbiota across ethnicities. HO, oral of the Han population; HG, gut of the Han population; TO, oral of the Tibetan population; TG, gut of the Tibetan population; MO, oral of the Hui population; MG, gut of the Hui population. Different lower-case letters indicate significant differences between the groups. (**b**) PCoA based on unweighted and weighted UniFrac distances comparing the bacterial community between the oral and gut microbiota.

Alpha diversity analyses using the Shannon index and observed species showed that, for the Han group, the oral microbial diversity was higher than in the gut microbiota. The Tibetan and Hui groups exhibited similar patterns of diversity (Figure 4a). We then investigated whether the alpha diversity of the oral microbiota was linked with that of the gut microbiota using a Spearman’s correlation analysis and found no significant relationship between the alpha diversity of the oral and gut microbiota (Supplementary Figure S4). For beta diversity, a PCoA based on unweighted and weighted UniFrac distances indicated a clear separation between the gut and oral microbiota although the oral microbes from the three ethnic groups were clustered together as were the gut microbiota from all groups (Figure 4b). The PERMANOVA analysis showed that the composition and structure of the oral and gut microbiota were significantly different across groups (*p* < 0.05).

**Figure 5:**
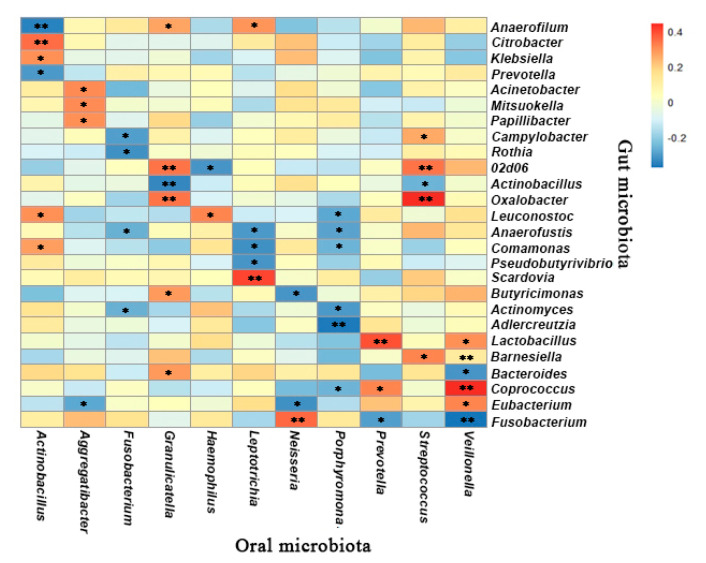
Genus level correlation heatmap between the oral microbiota and gut microbiota (* *p* < 0.05, ** *p* < 0.01; the *p* value was calculated using Pearson’s correlation).

A total of 123 oral bacterial taxa were significantly associated with 191 gut microbes with a correlation coefficient of >0.3 or <−0.3 and most were positively correlated. The correlation between the oral genera and gut genera with a relative abundance of >1% is presented in Figure 5. Oral *Prevotella* was positively correlated with intestinal *Coprococcus* and *Lactobacillus* (r = 0.31–0.39, *p* < 0.05). Oral *Streptococcus* showed a positive association with three microbes from the gut including *02d06*, *Barnesiella* and *Oxalobacter* (r = 0.32–0.44, *p* < 0.05). *Porphyromonas* from the oral microbiota was negatively related to *Actinomyces* and *Adlercreutzia* from the gut (r = −(0.31–0.37), *p* < 0.05). Oral *Neisseria* was positively associated with *Fusobacterium* from the gut (r = 0.35, *p* < 0.05) but inversely related with *Eubacterium* and *Butyricimonas* (r = −(0.31–0.32), *p* < 0.05). These results indicated that there were significant differences as well as correlations between the specific taxa within the oral and gut microbiota.

## 4. Discussion

In the present study, we analyzed the composition of the oral microbiota in Tibetan, Hui and Han school children living in the Qinghai–Tibetan Plateau. The most frequently detected phyla in the oral microbiota were *Bacteroidetes*, *Proteobacteria*, *Firmicutes* and *Fusobacteria*, cumulatively representing 94.54% of the sequences. These phyla were predominant in several previous studies on the oral microbiota of children and the elderly although different proportions were reported [30,31,32]. Notably, the levels of *Streptococcus*, *Acinetobacter*, *Moraxella*, *Porphyromonas* and *Anaerovorax* all varied significantly among the groups, which may have some relationship to oral health, given previous studies showing the differences in the prevalence of a few genera that were potentially correlated with conditions of oral disease [3,7,8]. While relative abundances for most of these genera were <5%, *Streptococcus* and *Porphyromonas* were found at substantially higher levels in our samples.

A few species such as *Streptococcus mutans* have been studied intensively for their potential roles in pathogenesis [33,34]; other *Streptococcal* species are frequently found among normal oral flora with no reported pathogenic effects. For example, Ling et al. [32] found that *Streptococcus* was the dominant genus in healthy Chinese children and adults while other studies reported that it was also the most abundant in both caries-free and caries lesion groups [35]. Li et al. also reported that *Streptococcus* could induce both pro- and anti-inflammatory responses [36]. Banas and Drake declared that the ecological balances and complexities within the entirety of the plaque microbiota should be emphasized other than the specific plaque hypothesis as an enrichment of *Streptococcus mutans* [37]. We found that *Streptococcus* had the lowest abundance in the Tibetan group although this condition did not indicate a difference in the likelihood of dental caries compared with other groups.

In contrast, the enrichment of *Porphyromonas* in the Han and Hui groups (school children) were reported in the present study, which was consistent with previous study of Han and Tibetan adults [38]. *Porphyromonas* is an important etiological agent of periodontal disease; the adaptability and survival of *Porphyromonas* in the oxidative microenvironment of the periodontal pocket are indispensable for survival and virulence and is modulated by multiple systems [39,40]. A study by Apatzidou et al. found that *Porphyromonas* was significantly higher among patients with peri-implantitis [41]. *Porphyromonas* was significantly more abundant in the saliva of patients with an oral squamous cell carcinoma and severe early childhood caries [42,43,44]. Therefore, the presence of *Porphyromonas* in a high abundance among the Han and Hui groups may be undesirable due to these reports suggesting its potential connection with a range of diseases although the subjects in this study had no obvious symptoms/signs of periodontitis. To date, no epidemiological investigation of oral diseases has been conducted for children on the Qinghai–Tibetan Plateau and future studies can build on this initial survey to explore correlations between the microbiota characterized in this study with an epidemiological investigation of specific diseases within these communities.

The alpha diversity indices showed that the observed species were not significantly different among groups although the Tibetan group had the lowest Shannon diversity (*p* < 0.01). However, it remains unclear whether the low community diversity is a consequence or cause of oral disease within this group, in light of previous studies that revealed that community diversity in the saliva of healthy populations was higher than that in populations with oral diseases such as dental caries, allergic diseases and obesity [42,45,46]. In the present study, a PCoA showed no significant differences in the beta diversity across the three ethnic groups, which suggested a high conservation of the oral microbiota although this finding may also be related to shared environmental and dietary factors. Previous studies using a principal component analysis (PCA) showed the separate clustering of Korean and Japanese individuals who also had notable differences in diet and environmental factors between them [47]. Nasidze et al. reported using a PCA to find a significant separation of the oral microbiota between the Batwa, a semi-nomadic hunter gatherer group of pygmies in Uganda, and two agricultural groups from Sierra Leone and the Democratic Republic of Congo thus illustrating the high impact of diet and environment on the structure of salivary microbiota [19].

While previous work has shown that dietary intake can be correlated with the prevalence of specific oral microbiota because food residues can provide nutrients that enrich certain taxa [48], it should be noted that a few claims reporting the influence of diet on the human salivary microbiota have been contradictory. For example, the oral microbiota of vegans and omnivores presented significant differences in composition at all taxonomic levels below the phylum level [17]. However, De Filippis et al. came to the opposite conclusion, finding that the diversity, community structure and taxonomy of salivary microbiota showed no differences among Italian vegans, lacto-ovo vegetarians and omnivores [49]. In the present study, a Pearson’s correlation showed that 13 genera were significantly associated with specific dietary components. In particular, vegetable consumption was positively correlated with *Bulleidia* and *Lactobacillus* but negatively correlated with *Bacteroides.* Previous works found that *Lactobacillus* and *Bulleidia* utilized carbohydrates to produce lactic acid or acetate, respectively [50,51,52]. In contrast, *Bacteroides* were linked to diets high in animal fat and protein [53]. We also found that the intake of sweets was negatively associated with *Clostridium*, *Enhydrobacter* and *Mycoplasma,* which provides a valuable extension to the work by Anderson et al. [54] in which an additional daily sucrose consumption led to significant increases in *Streptococcus* and decreases in *Haemophilus*, *Aggregatibacter*, *Prevotella* and *Porphyromonas* among oral biofilm-associated microbiota. Future work may explore whether these genera interact with each other cooperatively or in competition during enrichment by sucrose and, further, if these taxa are associated with disorders related to excessive sucrose consumption.

Previous work by our group has shown differences in the alpha diversity of the gut microbiota in school children from different ethnic groups [20]. In order to further explore the potential sources of these differences, in this work we performed a follow-up study to see if these differences were also found in the oral microbiota of the same children. Recently, Balakrishnan et al. reported an ethnicity-specific association in both the gut and oral microbial profiles between African American and European American children although no correlation analysis was conducted between the oral and gut microbiota within each group [55]. Khor et al. proposed two hypotheses to (at least partially) explain how oral bacteria may be transmitted to the lower digestive tract including the hematogenous route and the enteral route [56]. Given that the oral microbiotas are constantly swallowed along with saliva and delivered to the gut, we hypothesized that the oral microbiota would share a positive association with the gut microbiota. Surprisingly, we found that the oral microbiota had a higher alpha diversity than that of the gut and that the alpha diversity was not correlated between the oral and gut microbiota. Zhou et al. obtained similar results in the oral and gut communities of 279 healthy humans [57].

Moreover, a PCoA revealed that the oral and gut microbiota clustered separately with little or no overlap. We thus postulated that the following reasons could contribute to this phenomenon: (1) strong differences in the oral and intestinal environments likely select for a different microbial composition and (2) the gut microbiota, which undergoes a strong, long-term selection for the intestinal environment [58], can function as a competitive barrier against foreign bacteria from the mouth although, rarely, a few mouth-derived bacteria may overcome the inter-species competition and physical barriers (i.e., pH, anaerobic environment) to successfully colonize the gut [59].

Although we found a limited overlap in the distribution of their respective community members, we found that 123 oral microbes were correlated with 191 gut microbes, most of which shared a positive correlation, suggesting the contribution of oral microbes to shaping the gut microbiota composition. Schmidt reported that at least one in three oral microbial cells pass through the digestive tract to settle in the gut of healthy people and also suggested that the presence of oral commensals in the gut is a function of ectopic colonization, which can be potentially correlated with the risk of disease [59]. Chung et al. also found that a few oral microbiotas overlapped with the intestinal and pancreatic microbiota during a systemic disease such as pancreatic cancer and other gastrointestinal diseases although not in uniform abundance across environments [60].

In our work, *Prevotella* was the most abundant genus among the oral microbiota and was positively associated with *Coprococcus* and *Lactobacillus* from the gut. *Coprococcus* has been reported to potentially reduce liver inflammation and was observed to be less abundant in patients with simple steatosis and nonalcoholic steatohepatitis compared with healthy subjects [61,62]. *Lactobacillus,* as a probiotic, has been implicated in regulating the immune system function and has been tested as a treatment for gastrointestinal disease [63,64].

In addition, we found that *Porphyromonas* was negatively correlated with *Actinomyces* and *Adlercreutzia* in the gut. This finding supported those of Nakajima et al. [65] and Kato et al. [66] who reported that the oral administration of *Porphyromonas gingivalis* could significantly alter the gut microbiota composition in mice. This apparent interaction between *P. gingivalis* and the gut microbiota is plausible because *P. gingivalis* has been shown to be tolerant of a low pH (i.e., as in the stomach environment) and could therefore actively migrate and proliferate from the oral cavity through the intestinal tract [56,67]. Moreover, the oral administration of *P. gingivalis* in mice was also found to significantly exacerbate endotoxemia while reducing the transcription of genes such as *ZO-1*, *occludin* and *Tjp1* tight junction proteins in the small intestine. These rare but clear examples provide the strongest representative example of a species that connects the oral and gut microbiota in dysbiosis [65,66]. However, whether this regulation between the gut and oral microbiota is bi-directional and the underlying mechanism (e.g., metabolic by-products, direct colonization) by which oral communities influence the gut microbiota require closer examination. However, our evaluation of the oral microbiota was based on 60 saliva samples from three ethnic groups living in the high plateau and likely represents a conservative estimate for the wider population. Given our finding of significant correlations between the specific genera in the oral and gut communities, the full scale of the sample site as the tongue, gums and oral mucosa thus warrants a more comprehensive assessment and a future omics study will apply to a larger number of participants to verify the host variations and more closely scrutinize the relationship between the gut and oral microbiota as well as the effect of food and their regular consumption on the microbiota of individuals.

## 5. Conclusions

In conclusion, we found that the composition of the oral microbiota is generally conserved across Han, Hui and Tibetan ethnic groups in the Qinghai–Tibetan Plateau with little difference in diversity except for a significantly lower Alpha diversity for Tibetans compared with other groups. We also found that diet was associated with the presence of specific taxa in the oral microbiota of all groups and that although there was little or no overlap in the beta diversity between the oral and gut microbiota, we successfully identified significant correlations between the specific genera in the oral and gut communities. This work extends the findings of previous studies comparing differences in microbiotas from human populations living in close proximity and also provides a basis for further study exploring the interactions governing the tri-partite relationship between diet, the oral microbiota and the gut microbiota.

## Data Availability

The raw data of oral microbiota were deposited by accession number PRJEB34215 into the European Nucleotide Archive (http://www.ebi.ac.uk/ena/data/view/PRJEB34215). Other datasets presented in this article are available with requests directed to X.H., School of Public Health, Lanzhou University, Lanzhou China.

## Supplementary Materials

The following are available online at https://www.mdpi.com/article/10.3390/microorganisms9051030/s1, Figure S1: Sample rarefaction curves, Figure S2: Venn diagram at the OTU level (The overlaps represent the common taxa among different groups, and the non-overlapping portions represent unique taxa in each group), Figure S3: Composition of oral and gut microbiota. (a) Results at the phylum level. (b) Results at the genus level, Figure S4: Correlation between alpha diversity of oral and gut microbiota. (a) Shannon diversity. (b) Observed species, Table S1: Average daily food intake in the three ethnic groups, Table S2: Energy and mean percentage of energy from macro-nutrients among the three ethnic groups.
