# Supplementary material for: Oral Microbiota of Children Is Conserved across Han, Tibetan and Hui Groups and Is Correlated with Diet and Gut Microbiota
Source: Microorganisms. 2021 May 11;9(5):1030. doi: 10.3390/microorganisms9051030 (PMC8151815; doi:10.3390/microorganisms9051030)
Supplement: Supplementary file 1 [file microorganisms-09-01030-s001.zip › microorganisms-1159696-S/microorganisms-1159696-S1.pdf]

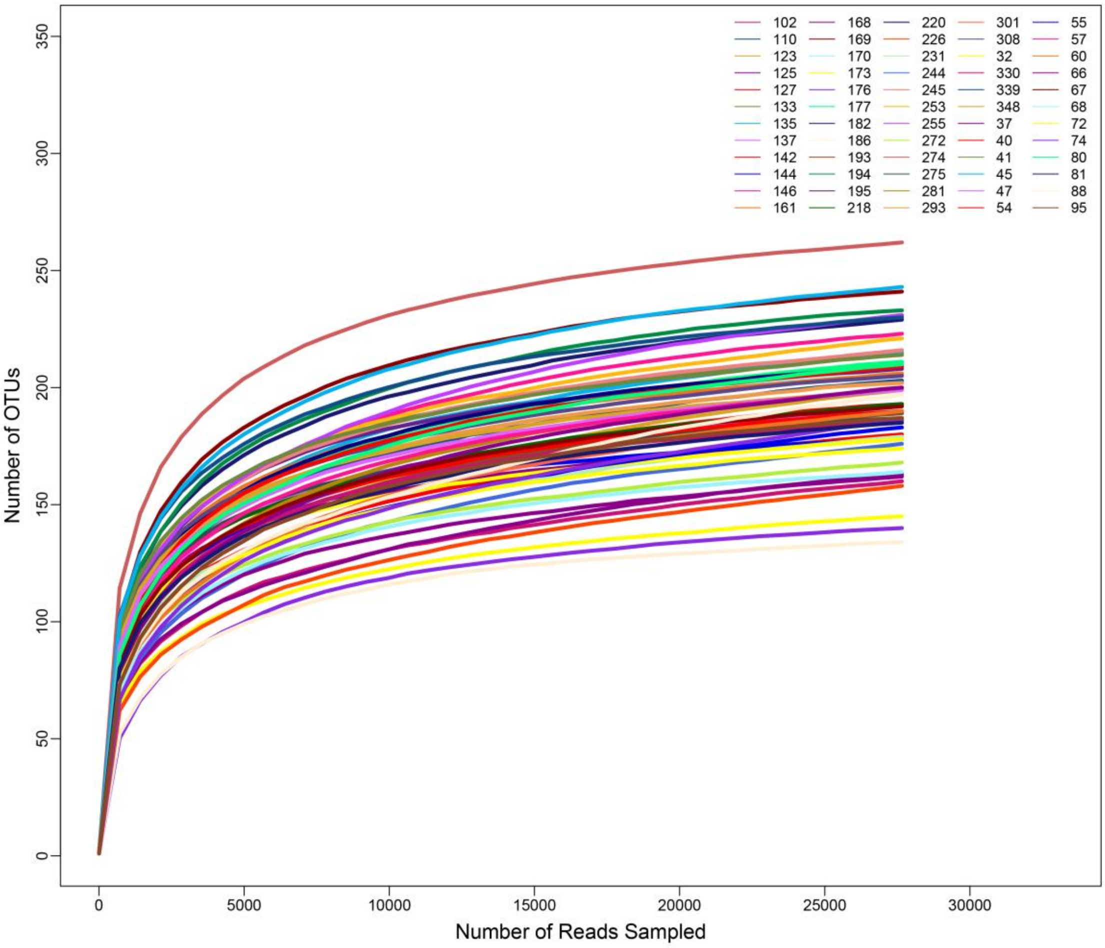

Supplementary Figure 1. Sample rarefaction curves.

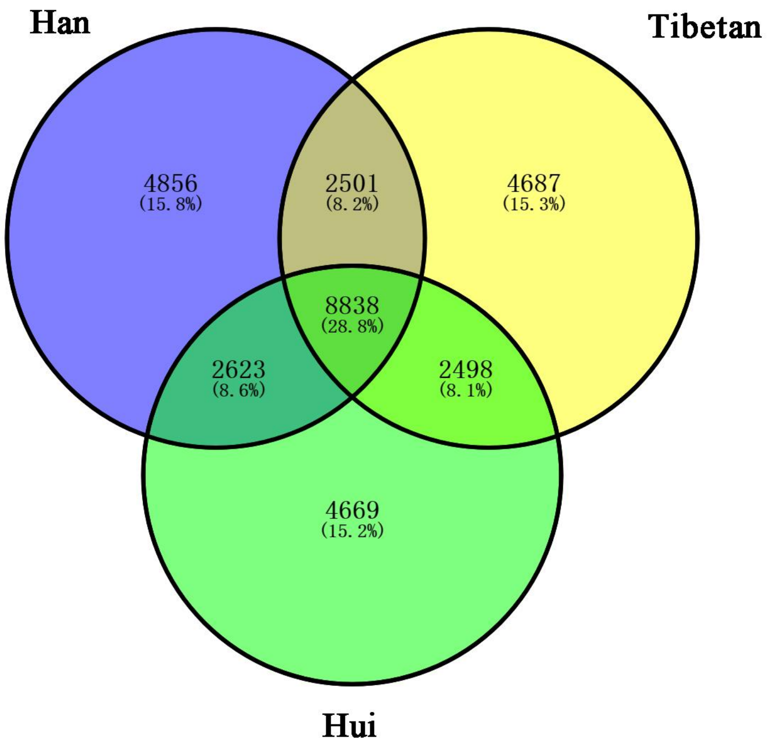

Supplementary Figure 2. Venn diagram at the OTU level. The overlaps represent the common taxa among different groups, and the non-overlapping portions represent unique taxa in each group.

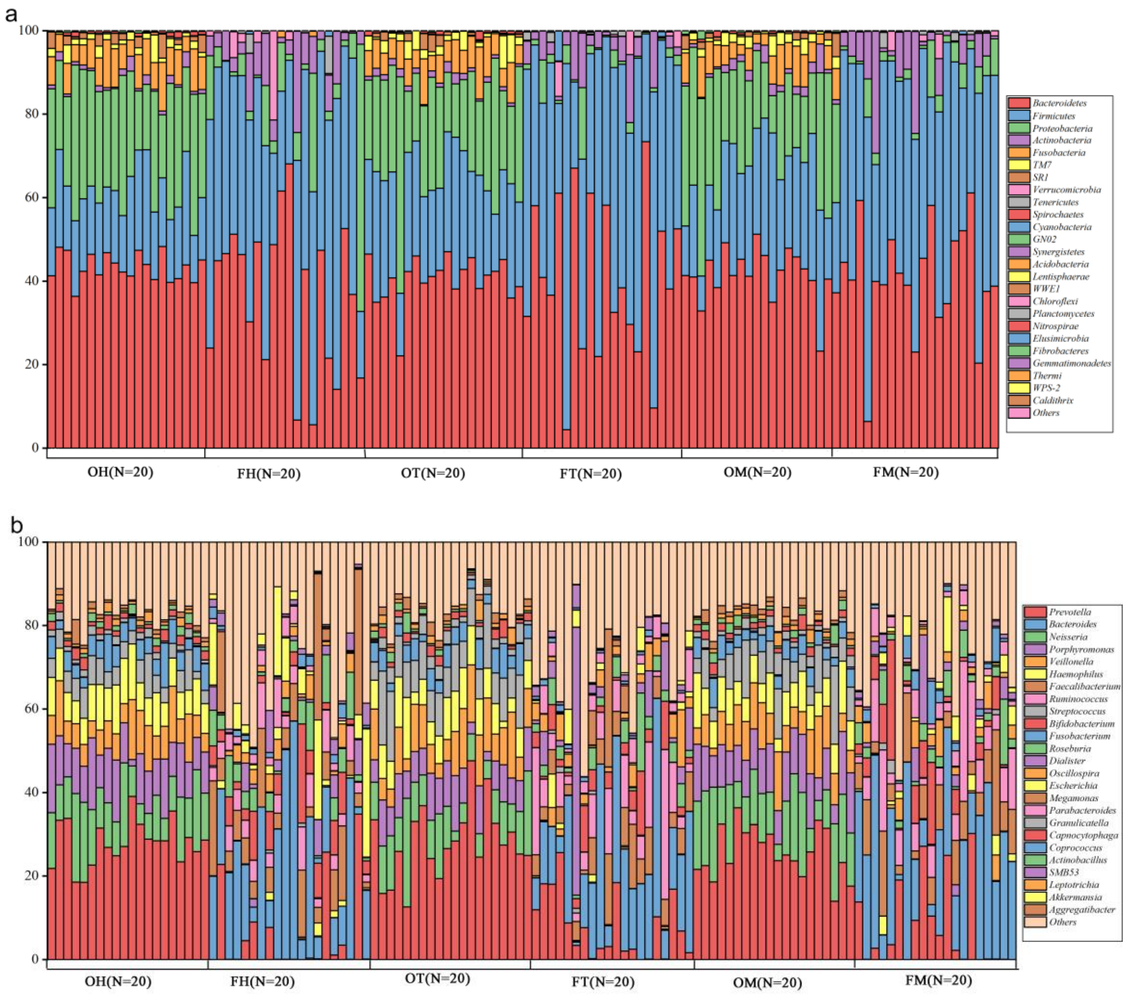

Supplementary Figure 3. Composition of oral and gut microbiota. (a) Results at the phylum level. (b) Results at the genus level.

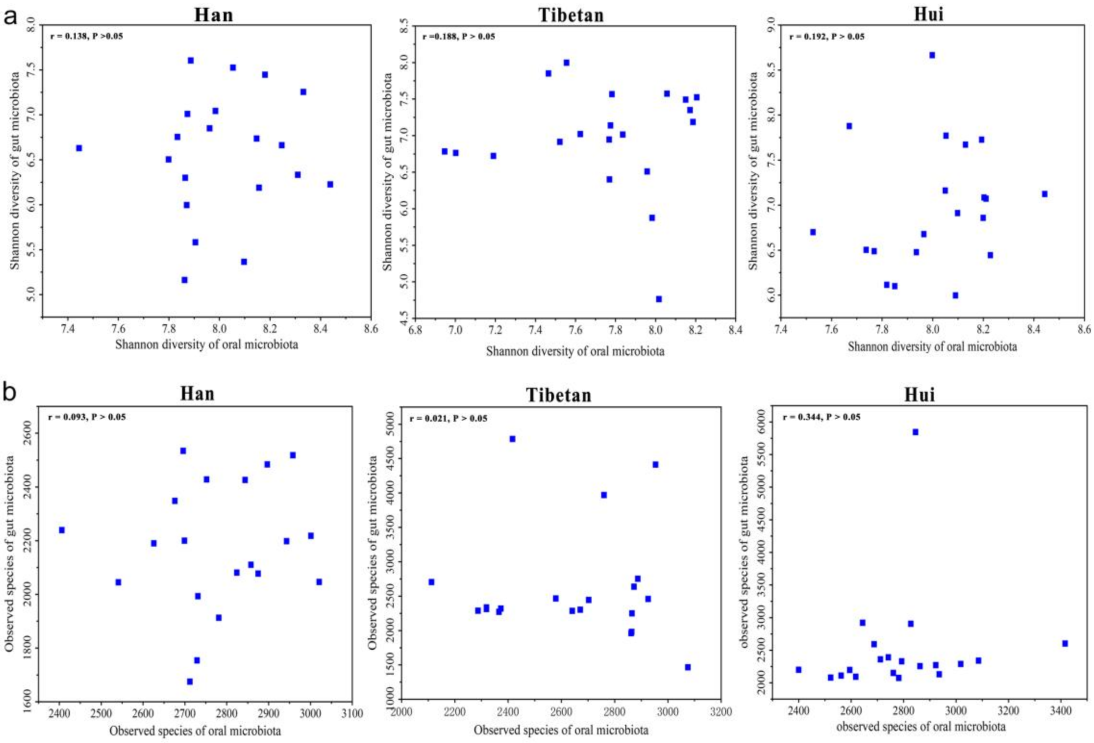

Supplementary Figure 4. Correlation between alpha diversity of oral and gut microbiota. (a) Shannon diversity. (b) Observed species.

Supplementary Table 1. Average daily food intake in the three ethnic groups.

Food species	Han (n = 20)	Tibetan (n = 20)	Hui (n = 20)
	Intake (g)	Intake (g)	Intake (g)
Grains	120.63(79.94 – 239.25)	137.75(70.00 – 320.25)	133.50(71.38 – 328.25)
Vegetables	114.14(40.06 – 262.38)	73.87(44.62 – 258.32)	93.45(28.78 – 223.45)
Fruits	157.71(58.09 – 269.64)	77.00(32.80 – 266.55)	92.33(36.46 – 246.41)
Poultry meat	6.61(2.41 – 12.30) *	0.22(0.00 – 6.00)	6.43(1.91 – 14.70) *
Livestock meat	17.22(4.15 – 23.92)	13.88(2.13 – 59.88)	12.58(3.31 – 69.00)
Seafood	3.75(1.15 – 13.22)	1.13(0.00 – 11.94)	1.03(0.00 – 9.60)
Milk	87.50(44.94 – 241.07)	91.00(50.00 – 317.75)	50.88(16.06 – 178.94)
Beans	7.75(0.00 – 44.80)	10.87(0.00 – 44.25)	3.63(0.00 – 49.00)
Eggs	8.52(0.00 – 16.10)	10.42(0.66 – 39.74)	8.90(0.10 – 30.09)
Nuts	13.87(1.37 – 34.86)	9.78(1.49 – 29.58)	2.90(0.63 – 12.17)
Condiments	106.63(25.24 – 216.76)	61.13(17.13 – 145.93)	63.50(14.57 – 203.69)
Sweets	11.13(3.13 – 27.94)	9.75(0.32 – 40.19)	7.25(2.69 – 46.88)

(Condiments included salt, vinegar, monosodium glutamate, pepper, soy sauce, and mustard. *compared with the Tibetan, $P < 0.05$; P value was calculated using Kruskal–Wallis rank sum test)

Supplementary Table 2. Energy and mean percentage of energy from macro-nutrients among the three ethnic groups.

Nutrient	Han (n = 20)	Tibetan (n = 20)	Hui (n = 20)
Energy (kcal)	1975.04 (981.77 – 2609.50)	2162.53 (1024.85 – 2861.62)	2034.99 (892.97 – 3842.89)
Protein (% energy)	13.45 (10.61 – 16.08)	13.13 (12.26 – 15.27)	13.13 (11.02 – 15.89)
Fat (% energy)	29.87 (25.66 – 40.27)	33.23 (28.85 – 39.08)	27.81 (15.87 – 36.60)
Carbohydrate (% energy)	55.32 (46.00 – 62.77)	51.55 (44.86 – 56.45)	61.46 (48.32 – 73.79)
