# Supplementary figures and images for: Oral Microbiota of Children Is Conserved across Han, Tibetan and Hui Groups and Is Correlated with Diet and Gut Microbiota
Source: Microorganisms. 2021 May 11;9(5):1030. doi: 10.3390/microorganisms9051030 (PMC8151815; doi:10.3390/microorganisms9051030)

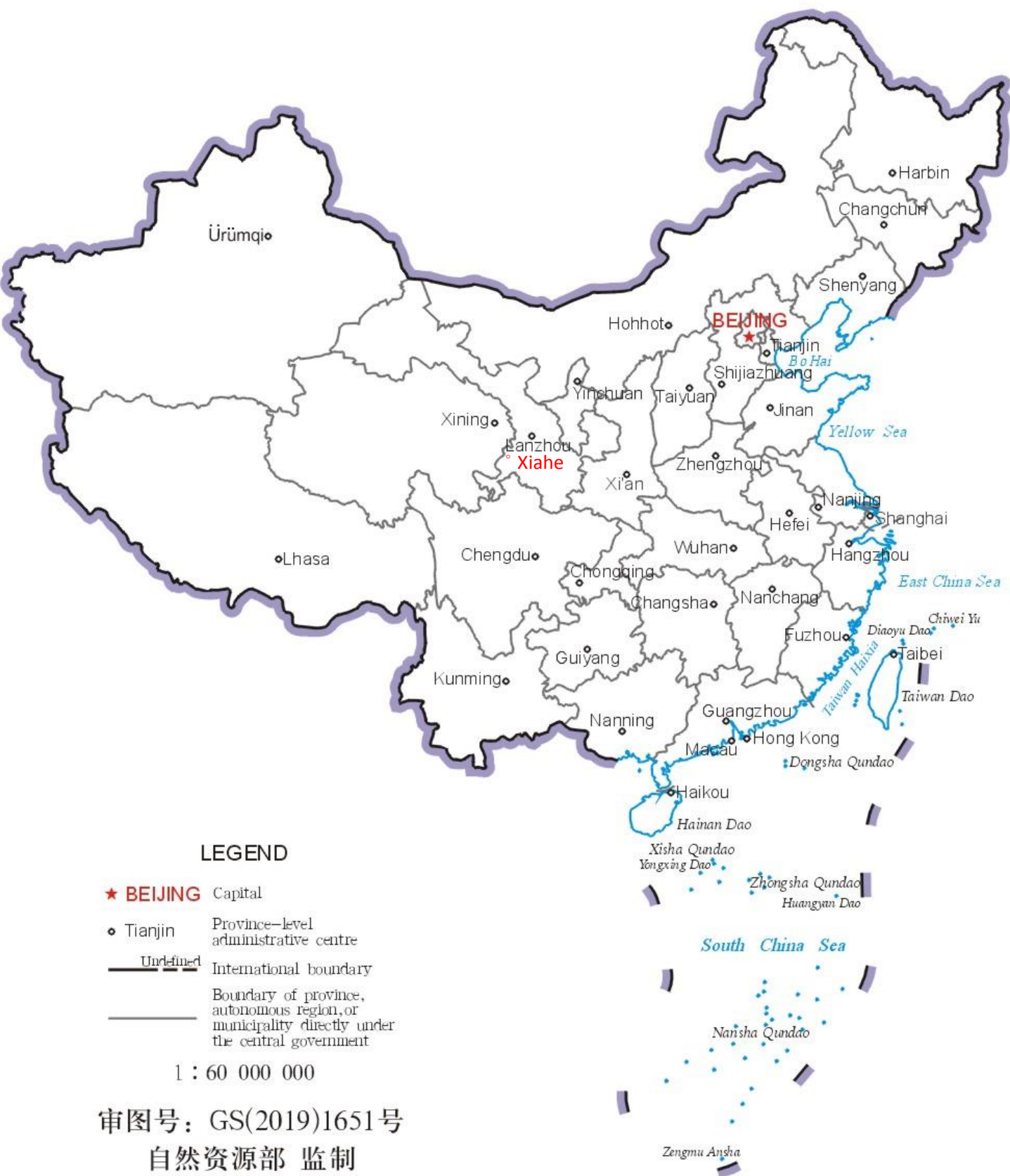

Supplement: Supplementary file 1 [file microorganisms-09-01030-s001.zip › microorganisms-1159696-S/microorganisms-1159696-S.pdf]
